# T-DM1 with concurrent radiotherapy in HER2-positive breast cancer: preclinical evaluation and mechanisms, prediction, and exploration of adverse effects

**DOI:** 10.1007/s12672-025-02239-2

**Published:** 2025-05-22

**Authors:** Guangmin Wan, Lu Yang, Quan Wang, Gang Xu

**Affiliations:** Department of Radiation Oncology, The Affiliated Cancer Hospital of Zhengzhou University & Henan Cancer Hospital, Zhengzhou, 450008 China

**Keywords:** T-DM1, Radiotherapy, HER-2, ADCs, Adverse effects

## Abstract

Human epidermal growth factor receptor 2 (HER-2) serves as a pivotal target for breast cancer treatment and a vital prognostic marker. Anti-HER-2 therapies, which are integral to the management of HER-2-positive breast cancer, including monoclonal antibodies (e.g., trastuzumab and pertuzumab), tyrosine kinase inhibitors (e.g., lapatinib and pyrotinib), and antibody–drug conjugates (ADCs) such as trastuzumab emtansine (T-DM1). ADCs consist of a monoclonal antibody, a linker, and a cytotoxic payload, engineered to deliver chemotherapy selectively to tumor cells, thereby reducing the systemic toxicity associated with traditional chemotherapy. T-DM1, a HER-2-targeting ADC, combines the humanized anti-HER-2 IgG1 trastuzumab with DM1, a cytotoxic agent that inhibits microtubule formation. T-DM1 has significantly enhanced the prognosis of HER-2-positive breast cancer patients who fail to achieve a pathological complete response or develop distant metastases after neoadjuvant trastuzumab and pertuzumab therapy. While the combination therapy of T-DM1 with radiotherapy demonstrates an acceptable safety profile overall, clinicians should remain vigilant regarding potential severe treatment-related toxicities that have been observed in specific clinical scenarios. Nevertheless, limited research exists regarding the adverse effects and mechanisms of T-DM1 in combination with radiotherapy. This review investigates preclinical studies on the interactions between T-DM1 and radiotherapy, investigates associated adverse effects and their underlying mechanisms, identifies predictive factors and prognostic implications, and explores potential therapeutic strategies involving the concurrent T-DM1 with radiotherapy.

## Background

**Fig. 1:**
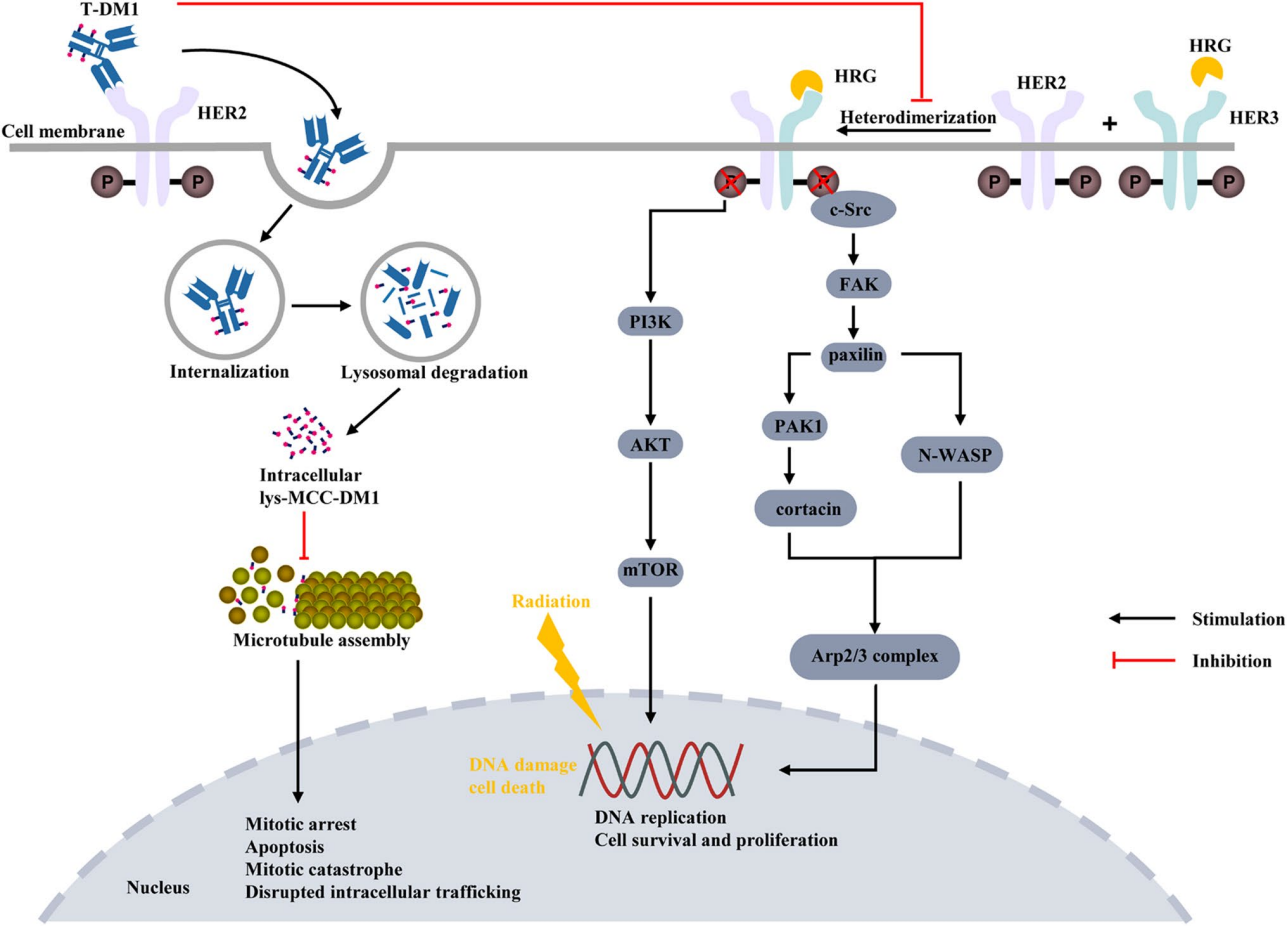
Mechanism of inhibition of HER-2-positive breast cancer cell proliferation by T-DM1 concurrent radiotherapy. *T-DM1* trastuzumab emtansine, *HRG* heregulin; *HER-2* human epidermal growth receptor factor 2; *Her-3* human epidermal growth receptor factor 3; *P* phosphorylation; *c-Src* proto-oncogene, non-receptor tyrosine kinase; *FAK* focal adhesion kinase; *PI3K* phosphoinositide 3-Kinase; *AKT* protein kinase B; *mTOR* mammalian target of rapamycin; *PAK1* activated kinase 1; *N-WASP* neural wiskott-aldrich syndrome protein; *Arp2/3* complex: actin-related protein 2/3 complex subunit 3

HER-2-positive breast cancer constitutes approximately 15–20% of all breast cancer subtypes and is characterized by high invasiveness and poor survival outcomes. Nevertheless, the anti-HER-2 therapies have markedly improved treatment outcomes for this subgroup [1, 2]. Neoadjuvant systemic therapy has demonstrated a high pathological complete response (pCR) rate in HER-2-positive breast cancer, increasing the feasibility of breast-conserving surgery and reducing the need for axillary lymph node dissection, while achieving survival outcomes comparable to those of adjuvant therapy [3–6]. However, several studies have revealed that patients with residual tumors following neoadjuvant therapy indicate poor prognoses, particularly in HER-2-positive breast cancer [7, 8]. The KATHERINE trial introduced T-DM1 as an intensified therapy for residual tumors, yielding promising results [9, 10]. Currently, ADCs targeting HER-2-positive breast cancer include T-DM1 and trastuzumab deruxtecan (T-DXd). By leveraging receptor-mediated endocytosis, T-DM1 (Fig. 1) selectively delivers the cytotoxic drug DM1 to HER-2-positive cells, sparing non-transformed cells and HER-2-negative tumor cell lines from cytotoxic effects [11]. T-DM1 has achieved significant milestones in the treatment of HER-2-positive breast cancer, and offered an effective targeted therapeutic option, which has been approved in metastatic HER-2-positive breast cancer following disease progression on trastuzumab therapy, as well as for adjuvant treatment in patients who fail to achieve pCR after neoadjuvant anti-Her-2 therapy [12]. Adjuvant radiotherapy remains the standard treatment for patients at high risk of regional recurrence after breast-conserving surgery or mastectomy [12]. Emerging evidence suggests that stereotactic ablative radiotherapy (SABR) significantly improves prognosis in patients with newly diagnosed oligometastatic or oligoprogressive disease [13, 14]. Consequently, the combination of T-DM1 with radiotherapy is now being employed in both early-stage and advanced metastatic breast cancer.

The waiting time for postmastectomy radiotherapy in breast cancer patients is closely associated with the risk of local recurrence. Delayed initiation of postmastectomy radiotherapy leads to an increased local recurrence rate, with studies showing that a 1-month delay raises the relative risk of local recurrence by 8% [15, 16]. Consequently, it is crucial to commence postoperative radiotherapy as promptly as possible to minimize the delays and the associated risk of recurrence. Gao et al. reported that earlier initiation of anti-HER-2 targeted therapy during neoadjuvant treatment for HER-2-positive breast cancer significantly enhances therapeutic efficacy [17]. For patients who fail to achieve pCR after neoadjuvant trastuzumab therapy, an overlap between anti-HER-2 treatment and radiotherapy becomes necessary. Several clinical trials have demonstrated the survival benefits of combining T-DM1 with radiotherapy [9, 10], the primary objective of this combination therapy is to achieve long-term symptom control and metastasis management; however, it may also lead to increased toxicity. Thus, the safety of T-DM1 in combination with radiotherapy has been a focus of attention [18]. Structurally, the thioether linker between trastuzumab and emtansine in T-DM1 is irreversible, requiring degradation to release its cytotoxic payload, which contributes to its specific toxicity profile [19]. Compared to other anti-HER-2 therapies, T-DM1 has more frequent adverse reactions, with hematologic toxicity and hepatotoxicity being the most common [10, 20, 21]. Local radiotherapy can suppress cardiopulmonary function and bone marrow hematopoiesis [22–24]. Natangelo et al. summarized the common adverse effects observed during concurrent T-DM1 and radiotherapy treatment for HER-2-positive breast cancer, including cardiotoxicity, pulmonary toxicity, dermatologic toxicity, bone marrow suppression, and brain injury [25]. Therefore, investigating the mechanisms, risk factors, prognostic implications, and optimal strategies for managing adverse effects arising from the concurrent T-DM1 with radiotherapy in HER-2-positive breast cancer will enable clinicians to better understand these interactions, implement interventions timely, and improve patient’s survival and quality of life ultimately.

## Preclinical study of concurrent radiotherapy with T-DM1

The expression level of HER-2 is closely associated with radioresistance in breast cancer, as confirmed in endogenous HER-2-positive breast cancer cells [26], Mignot et al. further demonstrated a clear linear relationship between radioresistance and the level of HER-2 expression in breast cancer cells [27]. Several in vitro studies have indicated that while T-DM1 combined with radiotherapy enhances radiosensitivity in HER-2-positive esophageal cancer and gastric cancer, it does not sensitize breast cancer cells to radiation, although a strictly additive effect is observed [27–29]. Söderlund et al. noted that the regulatory protein heregulin-β1 activates the PI3K/Akt pathway, thereby reducing radiation-induced apoptosis in BT-474 cells. Subsequent studies have found that binding of heregulin (HRG) induces HER3/HER2 heterodimerization and, consequently, phosphorylation of the Src/FAK/paxillin/PAK1-cortactin/N-WASP/Arp2/3 complex pathway, enhancing BC cell motility. T-DM1 can counteract the invasion-promoting effects of Heregulin on HER-2-positive breast cancer cells [30, 31], potentially providing a theoretical basis for the radiosensitizing effect of T-DM1 combined with radiotherapy in HER-2-positive breast cancer (Fig. 1). Therefore, further preclinical studies, particularly in vivo experiments, are needed to explore the potential radiosensitizing effects of T-DM1.

The rapid advancement of genomic science, particularly next-generation sequencing (NGS) technologies, has enabled fast, cost-effective, and accurate sequencing of the human genome, thereby enhancing its clinical applications in diagnostics and biomarker development [32, 33]. Gedik et al. discovered that transforming acidic coiled-coil containing protein 3 (TACC3) is overexpressed in T-DM1-resistant cells. In patients responsive to T-DM1, TACC3 protein expression is decreased, whereas non-responders exhibit increased TACC3 expression during T-DM1 treatment. In vaccine trials, in vivo inhibition of TACC3 triggered immunogenic cell death and enhanced the anti-tumor efficacy of T-DM1 by inducing dendritic cell maturation and increasing cytotoxic T-cell infiltration into the tumor microenvironment [34]. Additionally, a study on locally advanced rectal cancer found that knockout TACC3 enhanced the inhibition of HCT116 and SW480 cell proliferation and colony formation induced by irradiation, while increasing radiation-induced apoptosis. This highlights TACC3 as a significant factor influencing overall survival (OS) and progression-free survival (PFS) in rectal cancer [35]. Similarly, Sun et al. reported that patients with high TACC3 expression exhibited CD133⁺ stem cell characteristics, glioma plasticity, and shorter OS following chemotherapy or radiotherapy in glioma [36]. Moreover, TACC3 plays a critical role in regulating the biological behavior of liver cancer [37]. Therefore, TACC3 expression may serve as a potential biomarker for the efficacy of T-DM1 combined with radiotherapy in the treatment of HER-2-positive breast cancer.

## Mechanisms of adverse effects of concurrent radiotherapy with T-DM1

Common adverse reactions associated with the combination of T-DM1 and radiotherapy in the treatment of HER-2-positive breast cancer include radiation-induced brain necrosis, hematologic toxicity (particularly thrombocytopenia), skin damage, cardiotoxicity, and pulmonary toxicity [18, 25, 38]. Understanding these adverse reactions and their underlying mechanisms is critical for clinicians to implement timely interventions, minimize the occurrence rates of adverse events—especially severe ones—and prevent treatment interruptions caused by these side effects. Table 1 provides a summary of the adverse reactions observed with T-DM1 combined with radiotherapy.

**Table 1:** Clinical data on the application of T-DM1 combined with radiation therapy

Author, Year	No. of patients (lesions)	Site of radiation	Radiotherapy techniques	Median total dose and fractions (range)	RT timing with T-DM1 (range)	TRAE (No. of patients)
Carlson et al., 2014 [39]	7	Brain	SRS	20 Gy (16–24 Gy)/1f	Median 8.5 days (3 d–449 d) after radiotherapy	Radionecrosis (4)
Krop et al., 2015 [40]	39	Breast/chest wall + regional node	NR	NR	NR	Neutropenia (1) (grade 3) Lung toxicity (1) (grade 2)
Jacot et al., 2016 [41]	36	Brain	WBRT/Focal radiation therapy/SRS	NR	16.8 months (0.6–52.3 months) after radiotherapy	NR
Geraud et al., 2016 [42]	3	Bone	Hypofractionated radiotherapy	15 Gy /5f or 8 Gy/1f	D3–D7/NR	No
Geraud et al., 2017 [43]	4	Brain	SRS	NR	During the radiotherapy	Radionecrosis (2)
von Minckwitz et al., 2018 [10]	740	Breast/chest wall + regional node	NR	NR	NR	Platelet count decreased (211) Skin injury (188)
Stumpf et al., 2019 [44]	16	Brain	SRS	20 Gy (18–25 Gy)/1–5f	Within 4 weeks	Radionecrosis (6)
Corbin et al., 2020 [38]	1	Chest wall + regional lymph nodes	Proton therapy	50 Gy/25 f	During the radiotherapy	Radiodermatitis
Zolcsak et al., 2020 [45]	14	Breast/chest wall + regional node	Normfractionated irradiation	50 Gy/25 f	During the radiotherapy	Radiodermatitis (14) Cardiac toxicity (2) Thrombocytopenia (1)
Mills et al., 2021 [46]	19(lesions)	Brain	SRS/FSRT	21 Gy (14–24 Gy) or 25 Gy (20–30 Gy)/3–5f	During the radiotherapy	Radionecrosis (1)
Bellon et al., 2022 [47]	239	Breast/chest wall + regional node	Hypofractionated or conventional fractionation	≥ 2.5 Gy/f or 50–60.4 Gy or 45.0–50.4 Gy	12 weeks after the beginning of T-DM1	Radiodermatitis (208) Pneumonitis (4)
Dastgheyb et al., 2023 [48]	35	Breast/chest wall + regional node	Conventional fractionation	50 Gy (3–60.4 Gy)/10–33f	Within 14 days of the start of RT	Radiodermatitis (208)
Chun et al., 2024 [49]	39	Brain	SRS	EQD2: (mean ± SD)100.6 ± 28.4 Gy	Received T-DM1 within 1 year	Radionecrosis (12)
Koide et al., 2024 [50]	15	Brain	SRS/WBRT	18–40 Gy/1–10f 30 Gy/10f	9 days (interquartile range: 5–21 days)	Radionecrosis (1–5)

*T-DM1* trastuzumab emtansine, *SD* standard deviation, *FSRT* fractionated stereotactic radiation, *SRS* stereotactic radiosurgery, *EQD2* equivalent dose at 2 Gy, *WBRT* whole-brain radiotherapy, *TRAE* treatment-related adverse events

## Radionecrosis

**Fig. 2:**
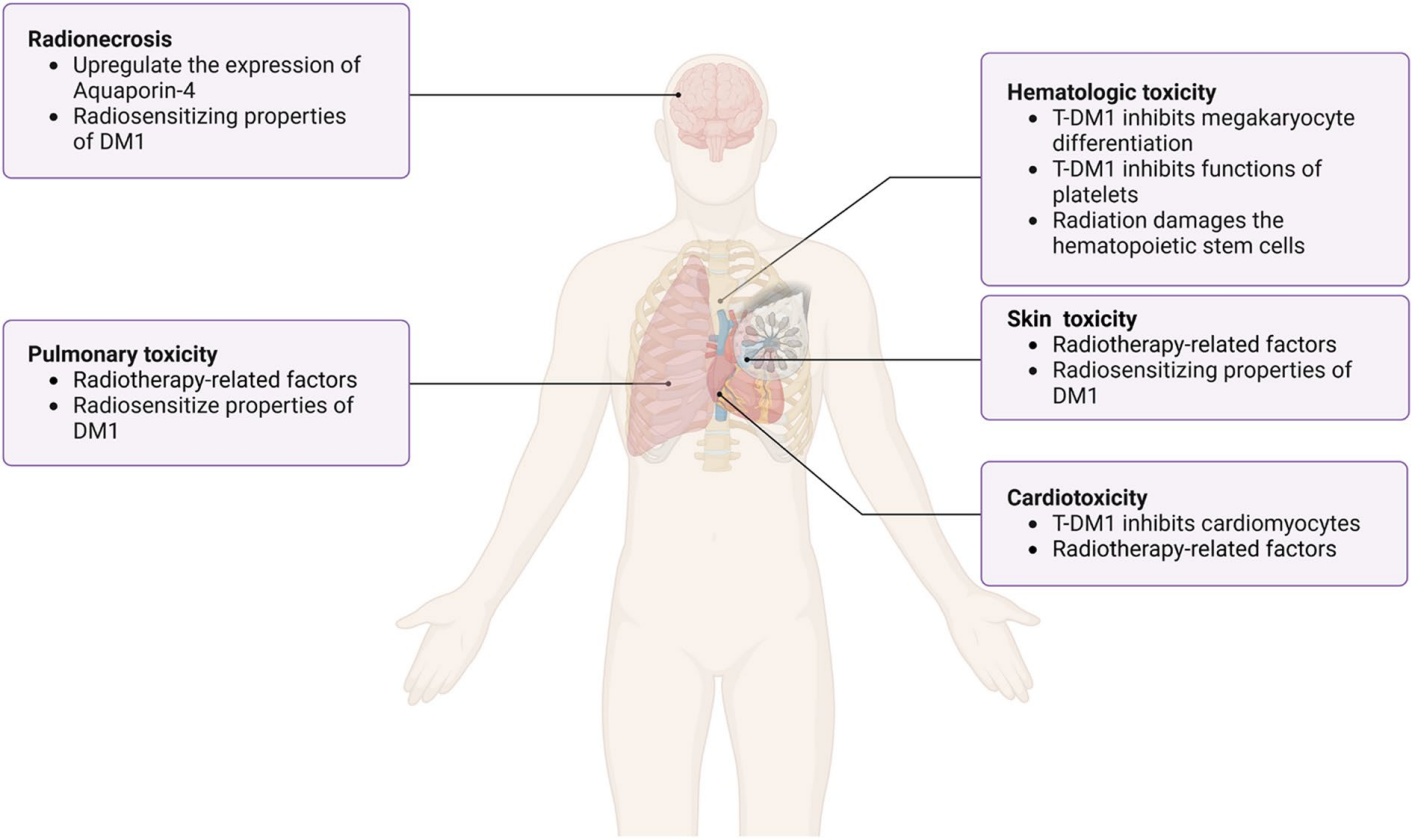
Common TRAEs of T-DM1 combined with radiotherapy and their mechanisms. *DM1* emtansine, *trae* treatment-related adverse events

Approximately 10%–30% of breast cancer patients develop brain metastases, with an especially high risk observed in HER-2-positive metastatic breast cancer patients, where the cumulative incidence reaches as high as 50%. Preclinical data suggest that the quantitative expression of HER-2 protein may be directly associated with the development of brain metastases [51–53]. Since the application of various HER-2-targeted therapies, overall treatment outcomes have significantly improved. Pyrotinib is a small-molecule tyrosine kinase inhibitor targeting HER1, HER2, and HER4. The phase II PERMEATE trial show the activity and safety of pyrotinib plus capecitabine in patients with HER2-positive breast cancer and brain metastases [54]. However, due to the high molecular weight of HER-2-targeted drugs and the low permeability of the blood–brain barrier, controlling intracranial disease remains a substantial challenge [55, 56]. Some studies have indicated that T-DM1 can penetrate the blood–brain barrier and reduce the incidence of brain metastases [57, 58]. Both whole-brain radiotherapy (WBRT) and stereotactic radiosurgery (SRS) can disrupt the integrity of the blood–brain barrier, thereby increasing the permeability of T-DM1. This enhanced permeability can persist for several days to months following fractionated or high-dose single radiation treatments [59–61]. Radiotherapy remains a cornerstone local treatment modality for central nervous system metastases. However, a series of studies have shown that combining T-DM1 with radiotherapy may increase the risk of radiation-induced brain necrosis [18, 44, 49, 62], Stumpf et al. reported that 39.1% of breast cancer patients with brain metastases who received T-DM1 developed radiation necrosis following combined SRS treatment [44]. Mechanistically, astrocytes can express HER-2, and T-DM1 may target reactive astrocytes surrounding brain metastases, leading to the upregulation of aquaporin-4 expression (Fig. 2), which may contribute to radiation necrosis induced by T-DM1 combined with SRS [44, 63]. Additionally, the radiosensitizing properties of DM1 may enhance T-DM1 cytotoxicity through increased uptake of DM1 by HER-2-positive astrocytes [44]. For any therapeutic agent, balancing proven efficacy with the associated targeted toxicity is critical to optimizing clinical outcomes. Therefore, heightened awareness of the potential interactions between T-DM1 and brain irradiation is essential.

T-DXd, a novel antibody–drug conjugate, combines a humanized HER2 monoclonal antibody with the topoisomerase I inhibitor deruxtecan. Preclinical studies in patient-derived xenograft models of HER2-positive breast cancer brain metastases demonstrated that T-DXd markedly reduces tumor volume and extends survival, even in T-DM1-resistant cases [64]. The DESTINY-BREAST03 Phase III trial further established T-DXd’s superiority over T-DM1, showing significantly improved intracranial response rates and PFS [65]. A Phase IIIb/IV trial further confirmed the substantial and durable systemic and intracranial activity of T-DXd, supporting its application in previously treated HER2-positive brain metastasis patients [66]. A recent real-world analysis evaluating T-DXd in HER2-positive breast cancer brain metastasis patients reported an intracranial objective response rate (iORR) of 59%, median intracranial progression-free survival (iPFS) of 15.6 months, and intracranial disease control rate (iDCR) of 94.9%, with a tolerable safety profile. Notably, 87.2% (34/39) of patients had undergone prior local interventions, predominantly SRS or WBRT, while 9 received concurrent SRS during T-DXd therapy [67]. Collectively, these findings highlight T-DXd’s clinical advantage over T-DM1 in HER2-positive breast cancer brain metastasis management, positioning it as a first-line therapeutic strategy for this challenging condition.

## Hematologic toxicity

The primary design objective of ADCs is to optimize the delivery of cytotoxic payloads to tumor tissues while minimizing their exposure to normal tissues, thereby maximizing therapeutic efficacy and minimizing toxicity. However, achieving this goal in tumor therapy remains challenging, as ADCs frequently cause severe hematologic toxicity. In adults, the bone marrow serves as the main organ of hematopoiesis, and radiotherapy for breast cancer unavoidably irradiates other normal organs such as the ribs and sternum, potentially damaging hematopoietic stem cells within the bone marrow. Radiation-induced damage to the bone marrow can directly or indirectly harm lymphocytes and hematopoietic stem cells, suppressing blood cell production [22]. Thrombocytopenia is a common and severe adverse reaction associated with T-DM1 treatment, characterized by transient onset on day 1 post-administration, reaching their nadir by approximately day 8, and recovering by day 15 [68, 69]. The DESTINY Breast 03 study and the KATHERINE study showed that the incidence of thrombocytopenia was 24.9%–28.5% [9, 70]. One study noted a high incidence of thrombocytopenia of 52.9% after T-DM1 application in the Chinese population, with a grade 3/4 thrombocytopenia rate of 21.6% [71]. Mechanistically (Fig. 2), thrombocytopenia results from impaired thrombopoiesis and reduced platelet survival in circulation [72, 73]. Mahapatra et al. reported that T-DM1 primarily inhibits the generation of megakaryocytes from human hematopoietic stem cells rather than directly targeting mature one[74]. Thon et al. demonstrated that T-DM1 permeates megakaryocytes and platelets through an HER2- and FcγRIIa receptor–independent pathway, impairing platelet production by inducing abnormal microtubule organization and inhibiting microtubule dynamics [75]. Subsequently, Uppal et al. discovered that T-DM1 does not directly impair platelet activation or aggregation but inhibits macrophage differentiation in an FcγRIIa-dependent, HER-2-independent manner [72]. Collectively, these studies suggest that T-DM1 internalization into megakaryocytes, independent of HER-2, contributes to thrombocytopenia. The discrepancies between these findings likely stem from differences in experimental methodologies and species variations. Zhao et al. proposed that FcγRIIa may not be the primary mediator of ADC internalization and differentiation impairment in megakaryocytes, suggesting that macrophage differentiation is inhibited through phagocytic-mediated internalization [76]. Additionally, Ansary et al. revealed that T-DM1 directly exerts toxic effects on the survival and aggregation functions of patient-derived platelets firstly [77], and provided new insights into the mechanisms underlying thrombocytopenia. Notably, the phase III MARIANNE trial observed thrombocytopenia in the T-DM1 and T-DM1 plus pertuzumab groups, while no such effect was detected in the trastuzumab plus paclitaxel group [78]. Based on these findings, we propose that T-DM1-induced thrombocytopenia may primarily result from the DM1 payload rather than the conjugated antibody. Furthermore, for certain ADCs, off-target damage to liver sinusoidal endothelial cells may contribute to acute thrombocytopenia [79]. The hematologic toxicity arising from the combination of radiotherapy and T-DM1 treatment needs further investigation, and uncovering its underlying mechanisms, which represents an important avenue for future research.

## Skin toxicity

Acute radiation dermatitis is a common toxic reaction associated with breast cancer radiotherapy, with nearly all patients experiencing grade 1–2 toxicity and more than half of post-mastectomy patients developing moist desquamation [48, 80]. However, skin damage caused by the combination of T-DM1 and radiotherapy has been underemphasized in previous studies, where the prevailing consensus is that the toxicity of T-DM1 combined with radiotherapy is no greater than that of radiotherapy alone. The KATHERINE trial reported that 25.4% of patients in the T-DM1 group experienced radiation-related skin injury of any grade [10]. Similarly, the ATEMPT trial reported a 33.9% incidence of grade 2 or higher skin toxicity [47]. However, neither trial provided detailed information about the radiotherapy modality or dose region. A recent meta-analysis suggested that the incidence of skin toxicity with T-DM1 concurrent radiotherapy was as low as 1% [18]. Corbin et al. described a case involving a patient who received proton therapy combined with T-DM1, with a prescribed radiation dose of 50 Gy/25 fractions over 30 days and no local boost, who developed grade 3 skin toxicity. They suggested that the KATHERINE trial may have underestimated the incidence of radiation dermatitis [38]. A subsequent small-sample study of 14 patients found that 12 developed grade 1 skin toxicity and 2 experienced grade 2 toxicity [45]. Additionally, Dastgheyb et al. described 35 patients who received a median radiation dose of 50 Gy (range: 30–60.4 Gy), with varying radiation plans and target areas. All patients developed at least grade 1 skin toxicity during treatment, with 23 reporting grade 2 toxicity and 3 developing grade 3 toxicity [48]. Various factors contribute to skin damage, including breast size, radiation dose, modality, fractionation, and skin care practices. Mechanistically, the primary mechanisms underlying radiation-induced skin injury are associated with DNA damage, excessive production of reactive oxygen species, metabolic alterations, protein turnover, cellular senescence, cell death, and vascular atrophy [81]. However, the mechanism behind skin damage occurring during TDMI concomitant radiotherapy for HER2-positive breast cancer requires further investigation.

## Pulmonary toxicity

In terms of lung toxicity, the likelihood of radiation-induced lung injury during concurrent T-DM1 treatment appears minimal [18, 82]. In the KATHERINE trial, the incidence of lung damage was 1.5% in the T-DM1 group compared to 0.7% in the trastuzumab group. Similar findings were reported in the ATEMPT trial [10, 47]. In a phase II clinical study by Krop et al., which included 116 patients, only 1 patient in the T-DM1 group developed grade 2 radiation pneumonitis [40]. Mechanistically (Fig. 2), DM1, a component of T-DM1, is a known radiation sensitizer and may increase the risk of radiation-related toxicity [44, 83]. Therefore, while the increased incidence of lung toxicity may appear incidental, the potential mechanism for T-DM1 to enhance radiation-induced damage demands further investigation.

## Cardiotoxicity

Preclinical studies have demonstrated that T-DM1 significantly reduces the viability of rat neonatal cardiomyocytes, human fetal cardiomyocytes, and adult-like cardiomyocytes, while also alter cell morphology, indicating inhibitory effects on cardiomyocytes (Fig. 2). Additionally, T-DM1 affects the contractile phenotype of adult-like cardiomyocytes in vitro and decreases the shortening fraction and ejection fraction in murine models [84]. Previous research suggests no significant difference in cardiac toxicity among different HER-2 targeted therapies [9, 10, 85]. However, data on T-DM1-induced cardiac toxicity remain limited. In the KATHERINE trial, the incidence of cardiac events in patients receiving T-DM1 was reported to be only 0.1% [10]. A retrospective single-center study of 14 patients receiving T-DM1 concurrently with radiotherapy found that the incidence and severity of acute cardiac toxicity were acceptable [45]. Traditionally, it has been believed that the heart has a high resistance to radiation, and symptoms of cardiac damage often exhibit a long latency period before becoming apparent, leading to insufficient attention to this issue [86]. Consequently, further prospective studies are critical to assess both acute and late radiation-induced toxicities, particularly cardiac toxicity, which may impact long-term survival.

Cardiac troponins are highly specific biomarkers of myocardial injury, and elevated troponin levels are independent predictors of cardiac toxicity [87, 88]. Antunac et al. reported that in patients with left-sided breast cancer receiving hyperfractionated radiotherapy concurrently with anti-HER-2 therapy, high-sensitivity cardiac troponin I levels were positively correlated with the radiation dose to cardiac substructures [89]. Although this study examined four combinations of anti-HER-2 therapies—trastuzumab, trastuzumab combined with pertuzumab, T-DM1, or trastuzumab and pertuzumab followed by T-DM1—no significant differences in troponin levels were observed between these treatments either before or during radiotherapy [89]. Mechanistically, radiation-induced heart disease (RIHD) is associated with endothelial cell injury, inflammatory responses, oxidative stress, mitochondrial and endoplasmic reticulum damage, cytokine release, calcium overload, and microRNA dysregulation [90]. With advancements in modern radiotherapy techniques, clinical practice must incorporate preventive measures, such as establishing dose constraints for cardiac substructures, employing proton therapy, implementing deep inspiration breath-hold techniques, and promoting lifestyle modifications. These approaches are particularly essential for high-risk populations with preexisting cardiovascular conditions to minimize the risk of cardiac toxicity effectively.

## Predictions of adverse effects of concurrent radiotherapy with T-DM1

Severe adverse reactions during treatment can lead to increased costs and treatment interruptions. Therefore, early identification of potential severe adverse reactions in HER-2 positive breast cancer treatment is essential for improving patient adherence and treatment outcomes. Identifying risk factors and implementing appropriate interventions can help minimize the occurrence of these adverse reactions.

For thrombocytopenia, dosage adjustment guidelines recommend discontinuing T-DM1 treatment if platelet counts drop to grade 3 or higher (below 50 × 10^9^/L) until platelet counts recover to grade 1 (above 75 × 10^9^/L). In cases of grade 4 thrombocytopenia (below 25 × 10^9^/L), treatment should be resumed at a reduced dose once recovery occurs. In the KATHERINE study, only 71.4% of patients completed T-DM1 treatment [10]. A meta-analysis involving 6188 patients with breast cancer, lung cancer, and gastrointestinal tumors indicated that Asian patients are at a higher risk of developing thrombocytopenia after T-DM1 treatment compared to non-Asian patients [91]. Modi et al. developed a clinical prediction model incorporating race and pre-treatment platelet counts, which effectively identified subgroups at risk of grade 3 thrombocytopenia following T-DM1 treatment [92]. Bender et al. constructed a platelet downward drift time curve, predicting that platelet counts would stabilize above grade 3 thrombocytopenia levels by the 8th treatment cycle [93]. Furthermore, a pharmacokinetic/pharmacodynamic model developed by Ait-Oudhia et al. demonstrated that a fourfold increase in T-DM1 dosage could elevate hematologic toxicity by one grade [94].

Following the recognition that a high body mass index (BMI) negatively impacts breast cancer prognosis [95], a retrospective study found that obese patients receiving T-DM1 treatment were more likely to experience high-grade adverse reactions and require dose adjustments compared to non-obese patients [96]. For patients with high BMI and larger breast size, the supine position during radiotherapy results in higher radiation doses to the heart and lungs, whereas prone-position radiotherapy can reduce the occurrence of adverse effects [97, 98]. Additionally, studies have shown that patients with larger breasts are four times more likely to develop moist desquamation compared to those with smaller breasts [99]. Sequential T-DM1 treatment following stereotactic radiosurgery, higher radiation doses, and a history of whole-brain radiotherapy have been identified as risk factors for radiation necrosis [49, 62]. Manus et al. were the first team to report predictive factors for neutropenia and thrombocytopenia, including concurrent chemotherapy and the volume of bone marrow irradiated [100]. A large-sample analysis involving 4055 patients found that radiation parameters, concomitant chemotherapy, and primary disease were associated with lymphocytopenia [101]. Takeda et al. established a nomogram to predict neutropenia, leukopenia, and anemia in patients undergoing radiotherapy. However, due to the small sample size, they were unable to develop a predictive model for thrombocytopenia [102]. In conclusion, factors such as race, drug dosage, treatment cycles, BMI, position fixing, and radiation dose significantly influence the occurrence of adverse reactions. Although there is no predictive models for adverse reactions during T-DM1 concurrent radiotherapy have been developed, the risk factors identified in these studies provide valuable insights for preventing adverse reactions in clinical practice.

## Impact of adverse effects of concurrent radiotherapy with T-DM1 on patient prognosis

Severe adverse reactions during treatment can delay or even interrupt subsequent therapy, thereby adversely affecting the prognosis of cancer patients. A study that first linked the systemic toxicity of T-DM1 to clinical outcomes analyzed 73 patients with advanced HER-2 positive breast cancer undergoing T-DM1 treatment. The analysis revealed that more severe systemic toxicity from T-DM1 was significantly associated with longer PFS [103]. A multicenter retrospective study examined the impact of thrombocytopenia on treatment discontinuation in patients with advanced HER-2 positive breast cancer receiving T-DM1. Among 138 patients who had undergone surgery or other local treatments such as radiotherapy, 39% experienced thrombocytopenia during treatment, with 66.7% developing early-onset thrombocytopenia. Multivariate analysis identified independent factors influencing treatment discontinuation time, including hormone receptor status, Eastern Cooperative Oncology Group (ECOG) performance status, and thrombocytopenia during treatment. Notably, patients with early-onset thrombocytopenia had a significantly longer treatment discontinuation time (17.3 months) compared to those without early-onset thrombocytopenia (7.6 months). Furthermore, patients with early-onset thrombocytopenia exhibited improved survival outcomes compared to those without thrombocytopenia [104]. Terrones et al. reported that patients who developed lymphocytopenia faced a higher risk of death within 1 year after radiotherapy compared to those without lymphocytopenia [105]. Additionally, a lower ratio of the minimum peripheral blood lymphocyte count during radiotherapy to the pre-radiotherapy lymphocyte count was associated with poorer prognosis in breast cancer patients [106]. With the increasing use of concurrent radiotherapy and T-DM1 in HER-2 positive breast cancer, the impact of treatment-related toxicities on prognosis warrants further investigation.

## Exploration of concurrent radiotherapy with T-DM1

The treatment of breast cancer brain metastases (BCBM) requires a multidisciplinary approach aimed at controlling metastatic lesions, alleviating symptoms, improving quality of life, and maximizing survival time. Local therapy remains the cornerstone of treatment, encompassing surgery, stereotactic radiosurgery, fractionated stereotactic radiotherapy (FSRT), and whole-brain radiotherapy [107].

The indications for SRS regarding the number of brain metastases and the optimal radiation dose remain topics of ongoing debate. Some studies suggest that SRS is the preferred treatment for up to four brain metastases with a maximum diameter of less than 3.5 cm [108]. Yamamoto et al. found no significant difference in median OS or distant brain recurrence rates between patients with 2–4 BCBM and those with 5–10 BCBM. Subsequent studies also reported no significant differences in complications between these groups [109, 110]. Additionally, other studies have demonstrated the feasibility of using SRS to treat up to 15 or even 20 metastatic lesions [111–113]. However, Routman et al. emphasized that the total volume of metastases is a more critical factor than the number of lesions when selecting SRS [114]. According to the EANO-ESMO clinical practice guidelines, the maximum tumor volume suitable for SRS is 15 cm^3^ [115]. Regarding the radiation dose, the Radiation Therapy Oncology Group (RTOG) conducted a study involving 156 patients with recurrent primary brain tumors or brain metastases. Their SRS dose recommendations (15–24 Gy) were based on the maximum tumor diameter to minimize the risk of radiation necrosis [116]. Most clinical guidelines similarly recommend prescribing doses in the range of 15–24 Gy, with the specific dose depending on tumor size and location [115]. However, some studies suggest that lower doses (up to 20 Gy) may be sufficient for local tumor control [117, 118]. Sahgal et al. proposed reducing the prescribed dose by 1–2 Gy in cases of multiple metastases, as the treatment of each lesion in SRS can influence surrounding tissues [119]. In the context of SRS combined with T-DM1 treatment for BCBM, Carlson et al. observed symptomatic radiation necrosis in four out of seven patients who received sequential T-DM1 following SRS. The median time from SRS to the initiation of T-DM1 treatment was 8.5 days (range: 3–449 days), with SRS doses ranging from 16 to 24 Gy and the maximum lesion size ranging from 0.9 to 4.5 cm^3^ [39]. A case report involving two patients found that both developed radiation necrosis 5 years after SRS followed by T-DM1. The first patient had an asymptomatic right parietal brain metastasis approximately 10 mm in diameter, treated initially with SRS (D95 = 25 Gy) and subsequently with local surgery and post-surgical stereotactic radiotherapy (SRT) (30 Gy/5 fractions) following recurrence. The second patient had an asymptomatic left temporal lobe metastasis approximately 8 mm in diameter, treated with SRS (D95 = 25 Gy) [120]. Sebastian et al. compared SRS techniques using Gamma Knife and linear accelerator (LINAC) technologies, showing that while survival rates were comparable, Gamma Knife SRS exhibited a higher rate of radiation necrosis [121]. Therefore, in addition to considering lesion number, volume, and radiation dose, the choice of irradiation technology should also be taken into account, as different techniques directly affect the irradiated volume and outcomes.

The timing of targeted therapies in combination with radiotherapy is a novel concept, and the optimal scheduling for their use remains undetermined [122]. The half-life of T-DM1 is approximately 4 days [123], for patients with early-stage breast cancer, T-DM1 is typically administered in 14 cycles (21 days per cycle) unless disease recurrence or uncontrollable toxicity occurs. For metastatic breast cancer, T-DM1 is continued until disease progression or intolerable toxicity develops. Consequently, the time interval between the initiation of radiotherapy and T-DM1 treatment may be insufficient to accurately reflect the toxicity associated with combination therapy. The temporal relationships between radiotherapy and T-DM1 administration was summarized in Table 1. Stumpf et al. defined synchronous stereotactic radiosurgery and T-DM1 administration as receiving T-DM1 within 4 weeks of SRS. They observed that patients who had received T-DM1 prior to SRS (77–131 days before) developed radiation necrosis 221–374 days post-treatment, while those who received T-DM1 after SRS (420–1426 days after) developed radiation necrosis 8–92 days post-treatment. For patients undergoing synchronous SRS and T-DM1, radiation necrosis occurred 16–529 days post-treatment [44]. Similarly, Koide et al. defined synchronous antibody–drug conjugates (ADCs) as ADC administration within 4 weeks before or after radiotherapy [50]. Other studies have considered T-DM1 administration during radiotherapy as synchronous therapy [43, 46]. A multicenter retrospective study conducted in Korea and Italy found that patients receiving T-DM1 either before or after SRS had a higher risk of developing radiation-induced brain necrosis within 1 year of SRS [49]. Dastgheyb et al. analyzed the impact of T-DM1 administration within 14 days of radiotherapy initiation on the incidence of radiation dermatitis [48]. In summary, for HER-2-positive breast cancer patients receiving T-DM1, particularly regarding its timing relative to SRS, reliable data remain scarce. The lack of consensus on the optimal timing of T-DM1 administration in combination with radiotherapy highlights an important area for future research.

## Conclusions

T-DM1 is widely used as adjuvant therapy for HER-2-positive breast cancer; however, its combination with radiotherapy can result in a range of adverse effects. Whether T-DM1 sensitizes HER-2-positive breast cancer to radiation remains inconclusive based on current preclinical studies. The advent of next-generation sequencing technology provides an opportunity to identify biomarkers associated with the therapeutic effects of synchronous T-DM1 and radiotherapy. Several randomized controlled trials have demonstrated the survival benefits of T-DM1 in the treatment of HER-2-positive breast cancer. While the combination of T-DM1 and radiotherapy appears generally safe, severe adverse reactions have been reported in certain cases. Therefore, elucidating the mechanisms underlying these adverse reactions in synchronous T-DM1 and radiotherapy is of critical importance. Despite progress, research on the mechanisms, prediction, and prognostic implications of these adverse reactions, as well as the optimal treatment regimens for combination therapy, remains insufficient. Based on previous studies [62, 124], future research on synchronous T-DM1 and radiotherapy may explore strategies such as low-dose T-DM1 radiation sensitization, low-dose radiotherapy, omission of radiotherapy, or the potential use of T-DM1 in combination with specific chemotherapeutic agents.

## Data Availability

No datasets were generated or analysed during the current study.
